# Complete chloroplast genome sequence of *Chrysosplenium macrophyllum* and *Chrysosplenium flagelliferum* (Saxifragaceae)

**DOI:** 10.1080/23802359.2020.1760152

**Published:** 2020-05-12

**Authors:** Wen-Jie Yan, Tian-Ge Yang, Rui Liao, Zhi-Hua Wu, Rui Qin, Hong Liu

**Affiliations:** a Hubei Provincial Key Laboratory for Protection and Application of Special Plant Germplasm in Wuling Area of China, Key Laboratory of State Ethnic Affairs Commission for Biological Technology, College of Life Science, South-Central University for Nationalities, Wuhan, China; b Beijing Union University, Beijing, China

**Keywords:** *Chrysosplenium*, Chloroplastgenome

## Abstract

The complete chloroplast genome of *Chrysosplenium macrophyllum* Oliv. and *Chrysosplenium flagelliferum* Fr. Schmidt. were reported in this study. The chloroplast genomes were 152,837 bp for *C. macrophyllum* and 151,679 bp for *C. flagelliferum*. LSC and SSC of 83,584 bp and 17,265 bp were separated by two IRs of 25,994 bp each in *C. macrophyllum*. While *C. flagelliferum* contained IRs of 25,973 bp, LSC of 82,772 bp and SSC of 16,961 bp, for a total 151,679 bp length. The chloroplast genome of *Chrysosplenium macrophyllum* contains 130 genes, including 85 protein-coding genes (78 PCG species), 8 ribosomal RNA genes (4 rRNA species), 37 transfer RNA genes (30 tRNA species). And the chloroplast genome of *Chrysosplenium flagelliferum* contains 130 genes, including 85 protein-coding genes (78 PCG species), 8 ribosomal RNA genes (4 rRNA species), 37 transfer RNA genes (30 tRNA species).

*Chrysosplenium* L. (Saxifragaceae) comprises about 65-70 species in the worldwide, mainly distributed in the northern hemisphere (Hara 1957; Soltis et al. 2001; Lan et al. 2018; Liao et al. 2019). They grow in moist, shaded valleys and mountain slopes (Kim et al. 2018). According to leaf arrangement, alternate or opposite, *Chrysosplenium* is divided into two subgenera: *Alternifolia* Franchet and *Oppositifolia* Franchet (Hara 1957). In this study, we report and characterize two chloroplasts genomes from two species: *Chrysosplenium macrophyllum* from subgenus *Alternifolia* and *Chrysosplenium flagelliferum* from subgenus *Oppositifolia*. Using these data, we reconstruct the phylogenetic tree of this genus to reveal the relationship and provide useful information for further study of *Chrysosplenium*.

The materials of *C. macrophyllum* and *C. flagelliferum* were collected from Hubei, China (BD2017030507344, N 30°44’04”, E 110°15’26”) and Tochigi-ken, Japan (RG2019032810002, N 36° 45′32″, E 139°36′01″). The voucher specimens were deposited at the Herbarium of South-Central University for Nationalities (HSN). The chloroplast DNA of *C. macrophyllum* was extracted by a high salt method (Shi et al. 2012) and sequenced using the PacBio II Platform at Frasergen (Wuhan China). The genome assembly was conducted using Canu-v1.5 (Koren et al. 2017). To discard the sequence of the nucleus, we aligned the contigs to the whole chloroplast data from NCBI. Then the draft chloroplast genome was polished with Arrow (SMRT link v6.0.0). However, the complete genomic DNA of *C. flagelliferum* was extracted using CTAB method and sequenced using the Illuminaplatform at Novogene Company (Beijing, China). After filtered the low-quality data and adaptors, the obtained clean data were aligned to *C. macrophyllum* with bwa-0.7.12. The aligned reads were then assembled with ABYSS-2.0.2 after the best Kmer was chosen with kmergenie. Then, connected the overlap and scaffolding again by SSPACE_Standard_v3.0. Finally, the gaps were filled by Sanger.

The complete chloroplast genome of *C. macrophyllum* (MK973001) was 152,837 bp in length and composed of two inverted repeats (IRs) of 25,995 bp which divide LSC of 83,583 bp and SSC of 17,264 bp, the average GC content was 37.46%. On the other hand, the complete chloroplast genome size of C. *flagelliferum* (MN729584) was 151,679 bp in length, the average GC content was 37.43%. Separating LSC of 82,771 bp and SSC of 16,960 bp, a pair of IRs was 25,974 bp long in each. The chloroplast genome of *Chrysosplenium macrophyllum* contains 130 genes, including 85 protein-coding genes, 8 ribosomal RNA genes, 37 transfer RNA genes. The chloroplast genome of *Chrysosplenium flagelliferum* contains 130 genes, including 85 protein-coding genes, 8 ribosomal RNA genes, 37 transfer RNA genes.

Phylogenetic analysis was performed using whole chloroplast coding sequences of *C. macrophyllum* and *C. flagelliferum*, combining with three species of Saxifragaceae, four species of Crassulaceae, four species of Paeoniaceae, two species of Hamamelidaceae, Iteaceae, Penthoraceae, and *Buxus microphylla* as outgroup. The phylogenetic relationships were reconstructed by means of maximum-likelihood (ML) with the model of GTR + F+R3. Based on the phylogenetic tree, we can see that *C. macrophyllum* and *C. flagelliferum* have a close relationship with *Bergenia scopulosa* (Figure 1).

**Figure 1.**
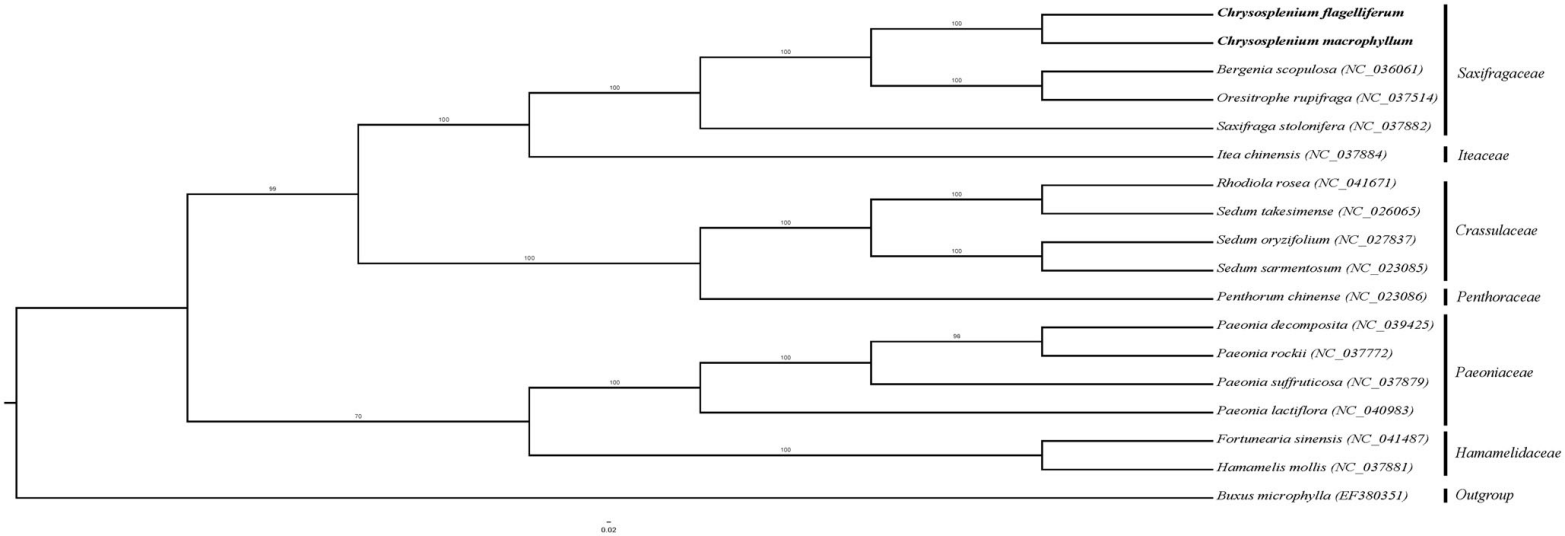
Maximum likelihood phylogenetic tree based on 18 complete chloroplast genomes. The number on each node indicates the bootstrap value. The bold part is C. macrophyllum and C. flagelliferum in this study.
